# Assessing Barriers to and Level of Adherence to Hypertension Therapy among Palestinians Living in the Gaza Strip: A Chance for Policy Innovation

**DOI:** 10.1155/2020/7650915

**Published:** 2020-09-24

**Authors:** Nasser Ibrahim Abu-El-Noor, Yousef Ibrahim Aljeesh, Bettina Bottcher, Mysoon Khalil Abu-El-Noor

**Affiliations:** ^1^Faculty of Nursing, Islamic University of Gaza, Gaza, State of Palestine; ^2^Faculty of Medicine, Islamic University of Gaza, Gaza, State of Palestine

## Abstract

**Introduction:**

Hypertension is a major health concern, especially in low-income countries. Nonadherence and poor or no persistence in adhering to hypertension treatment regimens result in uncontrolled high blood pressure, increasing rates of mortality and morbidity, and preventable healthcare costs. The aim of this study was to assess the level of adherence and barriers to treatment regimens among hypertensive patients living in the Gaza Strip, Palestine.

**Methods:**

A convenience sample of 648 participants completed the Hill-Bone Compliance to High Blood Pressure Therapy Scale. The great majority of participants (*n* = 521, 80.4%) was highly adherent to their treatment regimen, 123 participants (18.98%) were classified as moderately nonadherent, and 4 (0.62%) participants were classified as highly nonadherent to their hypertension treatment regimen. Participants of this study showed the highest adherence rate to the domain of medication adherence (mean of 1.42 out of 4) followed by appointment keeping (mean 1.8), while they were least adherent to diet (mean of 2.18). The greatest three barriers to adherence to the recommended treatment regimen reported by participants were inability to exercise, inability to resist fast and fried food, and inability to keep themselves away from salty foods.

**Conclusion:**

Overall adherence to medication in Gaza was surprisingly good in patients with a diagnosis of hypertension for at least one year. However, adherence to lifestyle advice or dietary regimes remains poor. A combination of interventions using low-cost mobile technology, combined with face-to-face interventions by healthcare professionals, can be applied to improve adherence to hypertension treatment regimens in order to reduce the consequences of uncontrolled blood pressure.

## 1. Introduction

High blood pressure (BP) is often asymptomatic and usually referred to as the silent killer. It is responsible for approximately half of the incidence of stroke and ischemic heart diseases, and it is the leading cause of mortality worldwide, posing a formidable challenge to healthcare [1–6]. Moreover, Cicero et al. [7] found that patients with uncontrolled BP have higher levels of serum uric acid, which contribute to increasing incidence of cardiovascular complications. Patients with poorly controlled BP were more likely to have multimorbidity [8] and a higher mortality rate [9]. There is a tendency for risk factor clustering among hypertensive patients [10] which contributes to increasing the number of morbidities as well as preventable healthcare costs.

Globally, hypertension is one of the most prevalent chronic diseases and one of the greatest public health concerns [11]. It was estimated that more than 1.3 billion individuals in the world have hypertension with about two-thirds of them occurring in low- and middle-income countries [12], and it was the cause of more than 7 million deaths annually [13]. According to the annual report published by the Palestinian Ministry of Health, cardiovascular diseases remain the leading cause of death among Palestinians, accounting for 30.6% of deaths recorded in 2016 with about 8% of the total number of deaths related directly to hypertension with a rate of 21.5/100,000 of the population [14]. This represents an increased rate of death (from 13 to 21.5/100,000 of the population), which highlights the urgent need for taking actions to reduce these numbers.

One goal of antihypertensive therapy is to achieve optimal BP control and reduce co-occurring chronic conditions [8]. This can be achieved through increasing the adherence to antihypertensive treatment regimens. Adherence to therapy was defined by the World Health Organization (2003, 3) as “the extent to which a person's behaviour, taking medication, following a diet, and/or executing lifestyle changes, corresponds with agreed recommendations from a healthcare provider.” According to Burnier and Egan [15], adherence includes three major components: initiation of therapy, the extent to which the patient adheres to the prescribed regimen and persistence or discontinuation, describing the continuing medication for more than one year. Although the management and control of hypertension reduce morbidity and mortality [9], the percentage of patients with controlled hypertension has been reported to vary between 5.4 and 58% worldwide [16]. In addition, among treated individuals, more than half of hypertensive patients do not have their BP under control, even in high-income countries with excellent healthcare systems [17].

Antihypertensive medications, along with lifestyle improvements, play important roles to achieve optimal BP control and reduce their complications [18–22]. Recently, evidence-based studies reflected that the use of digital medicine and mobile phone applications have improved the level of adherence to treatment among hypertensive patients [23, 24]. The benefits from patients' adherence to antihypertensive therapies and controlling blood pressure could be reflected by substantial reductions in the incidence of stroke, myocardial infarction, heart failure, and total mortality [25–27]. Lack of adherence with blood pressure-lowering medication is a major reason for poor control of hypertension [28]. On the contrary, several factors were reported to affect the lack of adherence to prescribed treatment regimens including beliefs about illness and treatment, forgetfulness, side effects of medications, complexity of treatment regimens, lack of knowledge regarding hypertension and its treatment, financial difficulties, psychological factors, social support, and poor quality of life [6, 29–32]. To facilitate healthy lifestyle choices and adherence to treatment, support of self-care in patients is essential [33]. A growing body of evidence examines interventions improving patient self-care and evaluating the current situation and factors influencing good or deficient self-care, which are important bases for developing efficiency of interventions [33, 34]. Therefore, studying barriers that affect patient adherence to treatment can be helpful for healthcare providers to overcome these barriers and improve adherence to treatment.

Only a few studies in Palestine examined the adherence of hypertensive patients to the treatment. These studies revealed a level of adherence ranging between 36.8% and 54.2% [35, 36]. Despite the fact that cardiovascular diseases are the most common cause of death in the Gaza Strip [14], no studies were found to explore barriers impacting the level of adherence to hypertension treatment regimens among Palestinian hypertensive patients. Assessing adherence and exploring barriers to hypertension treatment regimens among hypertensive patients are important in order to highlight possible solutions to overcome these obstacles, thus improving the level of adherence to treatment among this group of patients. This will be consequently reflected on reducing the number and severity of associated complications, number and length of hospitalizations, and their impact on quality of life of patients and reducing healthcare-associated costs. Therefore, this study aimed to assess the level of adherence and barriers to hypertension treatment regimens among hypertensive patients living in the Gaza Strip, Palestine.

## 2. Materials and Methods

### 2.1. Study Design

A cross-sectional descriptive design was used in this study.

### 2.2. Target Population, Setting, and Sampling

The target population included adults (over 18 years) who agreed to participate, had been diagnosed with hypertension for at least one year prior to the time of data collection, and received at least one antihypertensive agent. Hypertensive patients who met these criteria were included in the study. Patients who had other comorbidities were not excluded from this study. This helped to explore if the presence of comorbidity had an impact on the participants' level of adherence or not. On the contrary, patients who were under the age of 18, were diagnosed with hypertension for less than one year, or did not receive at least one antihypertensive drug were excluded from the study.

A convenience sample of 689 participants was recruited randomly from primary healthcare centers across the Gaza Strip. The purpose of the study and their involvement were explained to them before they were invited to participate. The sample size was calculated on the basis of the annual report from the Palestinian Ministry of Health [14], estimating the total number of hypertensive patients, who follow-up care at governmental primary health centers, to be 92,600. Using an online sample size calculator (http://www.raosoft.com/samplesize.html), with a 95% confidence level of precision, the required sample size was 383. The research team increased the sample size to cover for cases that receive their treatment for hypertension outside the governmental primary healthcare centers, in which their number is not known.

### 2.3. Instruments

The data collection instrument consisted of three parts. The first part contained questions regarding demographic data about the participants along with history of the disease and medications they received. The second part was the Hill-Bone Compliance to High Blood Pressure Therapy Scale (Hill-Bone CHBPTS), which was developed by Kim et al. in 2000 [37]. The Hill-Bone CHBPTS was chosen because it met the objectives of the study. It measures the level of patient adherence to the complete therapeutic regimen. Therefore, this scale was suitable for this study. Furthermore, it is a transcultural instrument and focuses on three behavioral domains of adherence that are critical for hypertension care and control which are adherence to medication, diet, and appointments.

The original questionnaire consists of 14 questions which fall into three categories: medication adherence, dietary regimen adherence, and medical appointment adherence. A fifteenth item, related to how often participants eat extra salty food such as pickles, was added to the questionnaire by the research team because salty foods, as well as adding extra salt to food, are important parts of the local diet. Each item was measured on a four-point Likert scale: never (1), occasionally (2), often (3), and always (4); the minimum and maximum possible scores are 15 and 60, respectively. While a lower score means a higher degree of adherence, a higher score means higher level of nonadherence to treatment regimens. Based upon the review of the literature, the researchers prepared a list of 17 items of possible barriers for adherence to hypertension treatment. Each item was measured on a five-point Likert scale in which 1 = strongly agree and 5 = strongly disagree.

The Hill-Bone CHBPTS was translated into Arabic to remove language barriers. This was done by three bilingual members of the research team. Then, face validity of the instrument was assessed by two bilingual healthcare professionals who reviewed the translation. The reviewers provided a few suggestions to improve the quality of the translation and to make it more user-friendly. The final version of the tool was modified accordingly and was sent to seven experts in the field to examine its content validity. After the validation of the instrument, it was pilot tests by 10 participants who were excluded from the study. Scale reliability of the translated instrument was assessed using Cronbach's alpha which revealed an acceptable value of 0.745.

### 2.4. Data Analysis

Statistical Package for Social Science (SPSS), version 22, was used to compute and analyze the data. All responses provided by participants were entered into a personal computer. The accuracy of the data entered into SPSS was ensured by double-checking of 70 completed questionnaires (which were randomly selected), and the data entered into the computer were compared with the original data. The researchers also checked that all data fell within the accurate range for each item prior to data analysis. Two questionnaires were eliminated from the study because they had more than five missing items. Missing values were replaced with the means for each item.

Data analysis procedures included basic descriptive statistics to describe the sample. Means and standard deviations were computed for continuous variables. Frequencies and percentages were calculated for categorical variables. ANOVA and *t*-test were used to compare means among different variables. A *P* value ≤0.05 was considered to be statistically significant.

### 2.5. Ethical Considerations

Ethical approval was obtained from the Helsinki Committee (a research ethics committee) in the Gaza Strip. Furthermore; the Ministry of Health and the Palestinian Medical Relief Society provided the research team permissions to conduct the study at their primary healthcare centers. Each potential participant was approached by one of the data collectors who explained the purpose of the research to him/her. Then, each participant was asked to sign informed consent detailing the purpose of the study, the voluntary nature of participation, and the confidentiality of the information gathered from each one. It was explained to participants that refusing to participate or withdrawing from the study would not affect their treatment plan at the primary healthcare center.

## 3. Results

A total of 689 questionnaires were collected, with only seven participants refusing to participate in the study. Of them, 41 questionnaires were eliminated (two had more than three missing variables, six did not mention for how long they had been diagnosed with hypertension, and 33 had been diagnosed with hypertension for a period of less than one year). The remaining 648 valid questionnaires were included in the analysis.

### 3.1. Sociodemographic Characteristics of the Participants

The age of participants ranged from 23 to 88 years with a mean of 59.0 (±11.49) years. Of them, 41.8% (*n* = 265) were 61 years old or older, 61.0% (*n* = 396) of participants were females, 70.3% (*n* = 509) were married, 11.8% (*n* = 75) were illiterate, and the majority (*n* = 325, 51.9%) had benefited from high school or a higher level of education. A large majority (*n* = 542, 83.8%) of participants were nonsmokers, and 65.4% (*n* = 424) reported to exercise for at least 30 minutes once a week (Table 1).

### 3.2. Adherence to Hypertension Therapy

In 353 (39.0%) participants, duration of being diagnosed with hypertension was between 1 and 5 years, and 28.4% (*n* = 184) had been diagnosed with hypertension for 6–10 years. Most participants (*n* = 289, 60.6%) had at least one other chronic disease with diabetes mellitus (*n* = 275) being the most common comorbidity. The great majority of participants (*n* = 420, 64.8%) had one prescribed antihypertensive medication, with amlodipine besylate being the most commonly prescribed antihypertensive drug (*n* = 299) followed by enalapril (*n* = 152) and atenolol (*n* = 142). Moreover, 127 (22.7%) participants were prescribed a low dose of aspirin, and 79 (12.2%) received a diuretic (Table 2). Critically, 68 participants (10.5%) did not know the name of their medication.

Participants of this study showed highest adherence rates to the domain of medication adherence with a mean total score of 1.42 out of 4, while they were least adherent to diet with a mean total score of 2.18 (Table 3). Within the medication adherence domain, participants showed the highest level of adherence to the item “How often do you take someone else's blood pressure pills” (mean: 1.16), indicating that they rarely never took someone else's medication. On the contrary, the lowest adherence was displayed to the item “Run out of blood pressure pills” with a mean of 1.89 (Table 4). In the diet adherence domain, the item “How often do you eat extra salty foods such as pickles and salty grounded red pepper” received the lowest mean by participants highlighting the low level of adherence to the recommendation of low salt intake (Table 4).

In general, the great majority of participants (*n* = 521, 80.4%) was highly adherent to their treatment regimen (had a total score of 15–29). However, 123 participants (18.98%) were classified as moderately nonadherent (scored 30–44), and 4 (0.62%) participants were classified as highly nonadherent (scored 40 or more) to their hypertension treatment regimen.

### 3.3. Variables That Influence Adherence to Hypertension Treatment

Interestingly, the overall adherence scores on the Hill-Bone CHBPTS correlated significantly and in a negative direction with age (*r* = −0.201, *P*=0.0001) and duration of diagnosis with hypertension (*r* = 0.089, *P*=0.023) (Table 5). This indicates that nonadherence decreased with age and duration of diagnosis with hypertension. On the contrary, no correlations were found with the number of comorbidities or the number of antihypertensive drugs prescribed.

Impact of different variables on the level of participants' adherence to the hypertension treatment regimen was measured by ANOVA and *t*-test. Most variables had no impact on the level of adherence (Table 6), except for smoking (*P*=0.019) and age (*P*=0.0001). Smokers (mean = 24.82 ± 6.31) were more adherent to antihypertension therapy than nonsmokers (27.46 ± 7.91), and younger participants (<30 years) were significantly more nonadherent to treatment than those in all other age groups, and adherence is shown to increase with decreasing age (Table 6).

Finally, the mean of the total score of the Hill-Bone CHBPTS and its subdomains was calculated according to the type of antihypertensive medication received by the participants (Table 7). The total score for the Hill-Bone CHBPTS ranged between 24.02 (valsartan) and 25.72 (losartan). The means for the diet domain ranged from 7.75 (valsartan) to 8.9 (losartan). The means for appointment keeping ranged from 3.41 (enalpril) to 4.32 (Co-Diovan), while the means for the medication adherence domain ranged from 12.32 (atenolol) to 12.75 (Co-Diovan).

Since many participants received two or more antihypertensive drugs at the same time as shown in Table 2, it was not possible to infer if taking a specific drug had an impact on adherence to hypertension treatment regimens. Therefore, independent *t*-test was used to compare between the means of participants who were taking a specific drug (for example, amlodipine besylate) and those who were not taking this drug (Table 8). Results revealed no statistically significant differences between participants who were taking any specific antihypertensive drug and those who were not taking that drug.

### 3.4. Barriers to Adherence to Antihypertensive Therapy

Barriers to adherence to hypertension therapy reported by participants are listed by decreasing strength in Table 9 with 11 barriers charting >2 points from a maximum of 5. The greatest three barriers reported by participants were “inability to exercise,” “inability to resist fast and fried food,” and “inability to keep themselves away from salty foods” (Table 9).

## 4. Discussion

The results of our study revealed that the overall adherence to hypertension treatment regimens was moderate to good with a score of 1.66, when one represents the best possible adherence rate, while four represents the worst. The rates of adherence to antihypertensive medications and appointment keeping were moderate to good with scores of 1.42 and 1.8, respectively. Participants had more difficulty with their adherence to dietary advice, such as reducing salty foods, with a score of 2.14, representing poor-to-moderate adherence. The overall adherence to the management of hypertension was found to get better with older age as well as increasing duration since the diagnosis of hypertension was first made. However, no impact on adherence scores was found to be exerted by comorbidities or number of antihypertensive agents. Interestingly, smokers showed significantly better adherence to medications than nonsmokers. From the 17 examined barriers to good adherence to antihypertensive treatment regimens, all were rated <3 from a maximum of 4, representing a moderate-to-weak strength of feeling about them. However, among the highest rated barriers to follow recommended treatment regimens were difficulties with resisting certain food choices, such as fried or salty foods, as already identified in the difficulty of adherence to the domain of dietary management, as well as inability to do regular exercise.

Adherence to management regimens of hypertension remains a global challenge with rates of adherence around 30%–50% in low-income countries and 50%–72% in high-income countries [17, 35, 38–41]. In this study, adherence to medication was reported to be surprisingly good compared to international as well as previous local studies that found poorer rates of adherence to hypertension treatment regimens of chronic diseases [35, 39, 40, 42–45]. One reason for this relatively good adherence rate, compared with other international and local studies, could be related to the fact that only patients who have had hypertension for more than one year were included in this study. Other studies showed that the highest discontinuation rates were found among patients who had been diagnosed for less than one year, with discontinuation rates of up to 50% within the first year of diagnosis [46, 47]. Another factor for the high adherence could be the fact that many patients in the Gaza Strip are not working, either due to age or unemployment, as the unemployment rate is as high as 52% [48] among Palestinians living in the Gaza Strip. Therefore, patients in Gaza are less distracted from taking medication on time. Furthermore, antihypertensive agents are offered free for patients registered as refugees and for a very minimal charge to all others with no additional fees charged for the clinic visit, removing the common barrier of medication cost [49].

As in numerous other studies, this study found adherence to lifestyle and dietary recommendations to be a greater challenge than adherence to taking medication [50, 51] with reducing salt intake and regular exercise posing the greatest challenges. In light of the fact that it is well known that adherence to management not only improves blood pressure control but also reduces cardiovascular morbidity and mortality, this is an important failing in modern healthcare and contributes substantially to globally high mortality rates associated with cardiovascular diseases [28, 52–54].

A multitude of studies have examined potential factors affecting medication adherence and found that sociodemographic factors are poor determinants of adherence with very little consistency in findings [55, 56]. Furthermore, such factors are mostly nonmodifiable, such as age or gender, and therefore impractical to target with interventions. Some progress has been made to improve adherence by modifying the medication type, dosing, and regimen, with simplified regimens including single-once a day medication promoting best adherence [47, 57–60], which are reflected in the current guidelines for treatment of hypertension [61]. Despite the lack of local or national guidelines, 64.8% of participants were also on a single medication, mostly one dose and well-tolerated types [47, 57, 58, 60]. However, no clear recommendations exist on further strategies to promote adherence.

Medication adherence is a dynamic process and can vary in individuals as well as populations, possibly increasing around clinic visits in individuals or decreasing with disease duration [47, 62]; on the contrary, this study found that adherence to hypertension treatment improved with age and duration of diagnosis which was similar to the results of a study in Pakistan [63]. Furthermore, assessment of adherence often overestimates actual adherence, especially using a self-assessment method, as in this study with 80.4% of participants rating themselves to be adherent to treatment. Factors contributing to such high ratings include improvement of adherence around clinic visits, recall bias, and social acceptability bias, with participants wanting to give the socially acceptable answers [39, 45, 62]. Therefore, triangulation of methods, employing two or more distinctly different ways to assess adherence, might generally give more accurate results. However, barriers to adherence might have to be examined by self-assessment and are important in planning strategies to further improve treatment adherence [64, 65]. In concordance with other studies, lifestyle factors, such as diet and exercise, were reported to be the most difficult to modify for individuals. Even in a low-resource setting, as in the current study, the greatest barriers were cost-neutral changes in the diet and engaging in more exercise with “the medication is too expensive” or poor availability of medication only ranking number 5 and 7, respectively. Therefore, it is not surprising that patient-focused interventions to improve medication adherence were found to be the most effective in a meta-analysis on medication adherence interventions [66]. This is also true for this low-resource setting, where most antihypertensive drugs are available free or for little payment to patients. However, patients only buy those drugs that are not available at the primary healthcare centers, and these are usually expensive for local people with the high rate of unemployment and the poor incomes in the Gaza Strip. Other reviews also confirmed that interventions targeting patient behaviors were more effective than those targeting provider behaviors [46, 67]. However, healthcare providers retain a key role in modifying patient behaviors. This can be done in various ways such as reminders, reviews, information, education, use of phone applications, and motivational interviewing, to name but a few of the many interventions studied [55, 67, 68]. One study from Gaza examining adherence to treatment regimens among 369 patients with type 2 diabetes identified negative health beliefs about the disease, the treatment, or both to be a powerful barrier to achieving good adherence to the management regimen [43]. Healthcare providers can substantially influence health beliefs and, therefore, also adherence [23, 69, 70]. This is reflected in the higher adherence rates of older patients and smokers in this study, who might receive more attention and education from healthcare providers due to their higher risk exposure. However, clinic visits in Gaza are often brief, and face-to-face contact with healthcare providers is usually short, as clinics are very busy with more than 100 visits in a morning session. As a result, clinic visits are often used to pick up prescribed medication with little opportunity for discussion of adherence problems and barriers; although brief interventions to motivate and educate patients are effective strategies to modify patient behaviors and improve their adherence levels [67]. The role of the clinicians has a key importance in this context and is often not fully recognized in low-income settings, such as in this study [33, 49, 65, 71–73]. Reinforcement of the importance of adherence to treatment can also be done via cell phone technology or eHealth methods, which has been shown to help in integrating pill taking into the daily routines of patients and thus improving their level of adherence [23, 74]. As these are relatively low-cost interventions, such ways can be sought to improve adherence in low-income settings also.

Another important factor influencing the effectiveness of interventions to improve the level of adherence to treatment regimens is the context in which they are applied, and their effectiveness varies from one setting to another and from one individual to another [75–77]. Therefore, continuous evaluation of interventions to improve adherence to treatment regimens is essential to ensure their ongoing effectiveness [78, 79]. For low-income settings, it is important to recognize the possibility, context, and work at improving adherence to treatment by using strategies that are low cost and feasible to be implemented such as cellphone technology, eHealth, or reminder screens in clinic waiting rooms as well as face-to-face interventions with healthcare professionals, when possible.

## 5. Strengths and Limitations

The Hill-Bone Compliance to High Blood Pressure Therapy Scale is a specific tool to address treatment for hypertension and has been validated in many languages, but this Arabic translation had not been validated before. Self-reporting of adherence to treatment regimens is prone to underestimation of the problem, and employing a second objective measurement of adherence could have made results more accurate and reliable. Furthermore, actual blood pressure control has not been measured in this study to see how adherence correlates with blood pressure control.

The sample of this study was a convenience sample, which might limit its generalizability. Due to the geopolitical isolation of the Gaza Strip, participants had been recruited from the Gaza Strip only; however, participants came from all geographical regions within the territory. Thus, while this study is representative of the local population and might add important points for low-income settings, it might not always be generalizable to different high-income settings.

## 6. Conclusion

Overall adherence to antihypertensive medication was surprisingly good in patients with a diagnosis of hypertension for over one year. However, adherence to lifestyle advice or dietary regimes remains poor. A combination of interventions using low-cost mobile technology, combined with face-to-face interventions by healthcare professionals, can be applied to improve this further. Monitoring and documentation of any interventions are essential to evaluate potential success or failure of such interventions and apply such lessons in the future.

## Figures and Tables

**Table 1:** Sociodemographic characteristics of the participants.

Variable	Frequency	Percentage
*Age (N = 634)*		
Mean (minimum/maximum)	59.0 (±11.49) years (23–88 years)	
≤30	9	1.4
31–40 years	31	4.9
41–50 years	95	15.0
51–60 years	234	36.9
61–70 years	172	27.1
>70 years	93	14.7

*Sex (N* *=* *649)*		
Males	253	39.0
Females	395	61.0

*Marital status (N = 642)*		
Married	509	79.3
Single	32	5.0
Widow	83	12.9
Divorced	18	2.8

*Level of education (N = 638)*		
None	75	11.8
Primary	107	16.8
Preparatory	131	20.5
High school	176	27.6
Diploma	53	8.3
Bachelor	89	13.9
Postgraduate studies	7	1.1

*Smoking (N = 647)*		
Yes	55	8.5
No	542	83.8
Previous smoker	50	7.7
Number of cigarettes	Range: (1–40) cigarettes/day	Mean: 13.9 (7.90)
Age started smoking	Range: (10–49) years	Mean: 20.4 (7.26)

*Smoking water pipe or vape (N = 647)*		
Yes	7	1.1
No	625	96.6
Previous smoker	15	2.3

*Exercise and walking (N = 648)*		
Yes	424	65.4
No	224	34.6
1-2 times a week	194	45.5
3-4 times a week	101	23.7
5–7 times a week	131	30.8

**Table 2:** Medical history of participants.

Variable	Frequency	Percentage

*Length for diagnoses with hypertension*	Range: 1–40 years	Mean: 9.77 (±7.48)
1–5 years	253	39.0
6–10 years	184	28.4
11–15 years	94	14.5
≥16 years	117	18.1

*Presence of comorbidities*		
Yes	395	61.5
No	247	109
One	301	38.5
Two	87	74.7
Three or more	15	21.6
Diabetes	275	3.7
Heart disease	123	
Other chronic diseases	109	

*Number of antihypertensive medications*		
One	420	64.8
Two	147	22.7
Three	13	2.0
Missing (or do not know the name of their drugs)	68	10.5

*List of antihypertensive medications*		
Amlodipine besylate	299	54.56
Enalapril	152	27.74
Losartan	31	5.66
Bisoprolol	106	19.34
Atenolol	142	25.91
Co-Diovan (valsartan/hydrochlorothiazide)	23	4.20
Valsartan	51	9.31
Other medications	51	9.31

*Adjunct medications*		
Diuretics	79	12.2
Low-dose aspirin	127	22.7

**Table 3:** Domains of the Hill-Bone Compliance to High Blood Pressure Therapy Scale.

	No. of items	Minimum	Maximum	Mean of the total score	SD	Mean (total score/no. of items)
Diet	4	4.00	16.00	8.47	3.07	2.18
Appointment keeping	2	2.00	8.00	3.6	1.69	1.8
Medication adherence	9	9.00	31.00	12.82	3.90	1.42
Total score	15	15.00	49.00	24.90	6.14	1.66

**Table 4:** Hill-Bone Compliance to High Blood Pressure Therapy Scale responses.

Question: How often do you	Response rate (frequency/%)				Mean (SD)
	None of the time	Some of the time	Most of the time	All of the time	
(1) Forget to take your HBP medicine?	443 (68.5)	132 (20.4)	53 (8.2)	19 (2.9)	1.46 (0.77)
(2) Decide not to take your HBP medicine?	501 (77.7)	102 (15.8)	26 (4.0)	16 (2.5)	1.31 (0.67)
(3) Eat salty food?	232 (36.1)	208 (32.4)	117 (18.2)	85 (13.2)	2.09 (1.03)
(4) Shake salt on your food before you eat it?	169 (26.3)	225 (35.0)	146 (22.7)	103 (16.0)	2.28 (1.03)
(5) Eat extra salty foods such as pickles and salty grounded red pepper?	213 (33.2)	271 (42.2)	83 (12.9)	75 (11.7)	2.03 (0.96)
(6) Eat fast food? (fat cook, chips, burgers)	177 (27.4)	302 (46.7)	114 (17.6)	53 (8.2)	2.07 (0.88)
(7) Get the next appointment before you leave the clinic?^*∗*^	371 (57.6)	77 (12.0)	102 (15.8)	94 (14.6)	1.87 (1.14)
(8) Miss scheduled appointments?	378 (58.7)	136 (21.1)	54 (8.4)	76 (11.8)	1.73 (1.04)
(9) Leave the dispensary without obtaining your prescribed pills? (due to long line, closure of clinic, forgot)	394 (61.5)	166 (25.9)	45 (7.0)	36 (5.6)	1.57 (0.85)
(10) Run out of blood pressure pills?	282 (43.8)	218 (33.9)	78 (12.1)	6 (10.2)	1.89 (0.98)
(11) Skip your blood pressure medicine 1–3 days before you go to the clinic?	489 (75.9)	106 (16.5)	35 (5.4)	14 (2.2)	1.34 (0.68)
(12) Miss taking your blood pressure pills when you feel better?	495 (77.0)	85 (13.2)	32 (5.0)	31 (4.8)	1.38 (0.79)
(13) Miss taking your blood pressure pills when you feel sick?	497 (77.3)	118 (18.4)	13 (2.0)	15 (2.3)	1.29 (0.62)
(14) Take someone else's blood pressure pills?	578 (89.6)	40 (6.2)	20 (3.1)	7 (1.1)	1.16 (0.51)
(15) Miss taking your blood pressure pills when you care less?	457 (70.7)	121 (18.7)	45 (7.0)	23 (3.6)	1.43 (0.77)

^*∗*^Reverse coding/scoring.

**Table 5:** Correlation of the total HILL-BONE CHBPTS score with variables of hypertensive patients.

Variables	Correlation (*r*)	*P* value
Age	−0.201^*∗∗*^	0.0001
Duration of diagnosis with hypertension	−0.089^*∗*^	0.023
Number of comorbidities	0.028	0.478
Number of antihypertensive drugs	−0.012	0.773

^*∗*^Correlation is significant at the 0.05 level. ^*∗∗*^Correlation is significant at the 0.01 level.

**Table 6:** Impact of demographic variables on compliance.

	Mean	SD	*P* value
*Sex*			

Males	24.94	6.37	0.899
Females	24.87	6.0	

*Age*			0.0001

≤30	30.49	10.35
31–40 years	27.56	7.49
41–50 years	26.10	6.59
51–60 years	25.25	5.94
61–70 years	23.48	5.30
>70 years	23.71	5.50

*Level of education*			

None	25.00	6.05	0.229
Primary	25.67	6.91
Preparatory	25.16	5.50
High school	24.98	6.46
Diploma	24.24	6.40
Bachelor	23.66	5.32
Postgraduate studies	27.29	5.09

*Marital status (N = 642)*			0.996

Married	24.89	6.22
Single	24.89	6.73
Widow	24.91	5.76
Divorced	25.26	5.87

*Smoking*			0.019

Yes	27.46	7.91
No	24.82	5.91

*Exercise*			0.452

Yes	25.03	6.01
No	24.64	6.39

*Presence of comorbidity*			0.092

Yes	24.87	6.07
No	24.96	6.31

*Number of antihypertensive medications*			0.695
One	25.09	5.94	
Two	24.70	6.75	
Three	25.92	4.62	

**Table 7:** Scores of the HILL-BONE CHBPTS and its subdomains according to the antihypertensive drug.

	Diet	Appointment keeping	Medication adherence	Total score
Amlodipine besylate	8.40 (±3.40)	3.62 (±1.68)	12.65 (±3.85)	24.66 (±5.98)
Enalpril	8.83 (±2.93)	3.41 (±1.61)	13.27 (±4.53)	25.50 (±6.84)
Losartan	8.90 (±3.35)	3.77 (±1.746)	13.06 (±4.17)	25.74 (±7.02)
Bisoprolol	8.65 (±3.00)	3.75 (±1.52)	12.85 (±4.1)	25.25 (±5.94)
Atenolol	7.83 (±3.21)	3.93 (±1.70)	12.32 (±3.45)	24.08 (±6.19)
Co-Diovan	8.41 (±3.26)	4.32 (±1.62)	12.75 (±3.99)	25.48 (±5.62)
Valsartan	7.75 (±2.80)	3.90 (±1.86)	12.37 (±3.44)	24.02 (±5.54)
Other medications	8.70 (±2.57)	3.43 (±1.65)	13.43 (±4.55)	25.55 (±6.45)

**Table 8:** Total score of the Hill-Bone CHBPTS according to the type of the antihypertensive drug.

Drug		Mean	SD	*P* value
Amlodipine besylate	Yes	24.66	5.98	0.352
	No	25.11	6.27	

Enalpril	Yes	25.50	6.84	0.201
	No	24.71	5.91	

Losartan	Yes	25.74	7.02	0.436
	No	24.86	6.10	

Bisoprolol	Yes	25.25	5.94	0.507
	No	24.83	6.18	

Atenolol	Yes	24.08	6.19	0.378
	No	24.96	6.14	

Co-Diovan	Yes	25.48	5.62	0.627
	No	24.88	0.25	

Valsartan	Yes	24.02	5.54	0.247
	No	24.97	6.19	

Other medications	Yes	25.55	6.45	0.477
	No	24.85	6.12	

**Table 9:** Reported barriers to adherence to hypertension therapy.

Barrier	Mean	SD
(1) I am unable to do exercise	2.716	1.774
(2) I like fast and fried food	2.567	1.440
(3) I like salty foods (I cannot keep myself away from salty foods)	2.434	1.520
(4) In general, I do not like medications	2.423	1.517
(5) My medication is too expensive	2.370	1.540
(6) I have to take too many medications every day	2.346	1.525
(7) My medication is not available	2.095	1.454
(8) I experience side effects from medication	2.088	1.416
(9) I have no time for regular exercise in my life	2.017	1.537
(10) Lack of motivation as there is no cure	2.013	1.392
(11) I am not interested in doing exercise	2.002	1.529
(12) I find it difficult to follow my treatment regime	1.997	1.252
(13) I forget to take my medication	1.956	1.338
(14) I do not want to take my medication	1.953	1.347
(15) I am not able to go to the clinic to get my medication	1.911	1.586
(16) I am not interested in stopping smoking	0.363	1.110
(17) I would like to stop smoking, but find it difficult	0.362	1.082

## Data Availability

The data used to support the findings of this study are available from the corresponding author upon request.
